# Biomedical Informatics and Granularity

**DOI:** 10.1002/cfg.429

**Published:** 2004

**Authors:** Anand Kumar, Barry Smith, Daniel D. Novotny

**Affiliations:** 1 IFOMIS University of Saarland Postbox 151150 Saarbrücken D-66041 Germany; 2 Department of Philosophy University at Buffalo NY USA

## Abstract

An explicit formal-ontological representation of entities existing at multiple levels of granularity is an urgent requirement for biomedical information processing. We discuss some fundamental principles which can form a basis for such a representation. We also comment on some of the implicit treatments of granularity in currently available ontologies and terminologies (GO, FMA, SNOMED CT).


**Comparative and Functional Genomics**
*Comp Funct Genom* 2004; **5**: 501–508.
Published online in Wiley InterScience (www.interscience.wiley.com). **DOI:** 10.1002/cfg.429

**Conference Paper**

### Biomedical informatics and granularity

Anand Kumar[1]*, Barry Smith[1,2] and Daniel D. Novotny[1,2]
[1] *IFOMIS, University of Saarland, Saarbrücken, Germany*
[2] *Department of Philosophy, University at Buffalo, NY, USA*

**Correspondence to:
Anand Kumar, IFOMIS, University of Saarland, Postbox 151150, D-66041 Saarbrücken, Germany.
E-mail: akumar@ifomis.uni-saarland.de*

Received: 4 October 2004
Revised: 12 October 2004
Accepted: 13 October 2004

### Abstract

**An explicit formal-ontological representation of entities existing at multiple levels of granularity is an urgent requirement for biomedical information processing. We discuss some fundamental principles which can form a basis for such a representation. We also comment on some of the implicit treatments of granularity in currently available ontologies and terminologies (GO, FMA, SNOMED CT). Copyright © 2004 John Wiley & Sons, Ltd.**

**Keywords: clinical bioinformatics; medical informatics; life sciences data integration; ontology; knowledge representation; levels of granularity**

Organisms, including human beings, consist of a variety of anatomical structures which exist at different levels of granularity (also called 'levels of complexity' or 'levels of biological organization'). Analogous levels of granularity can be distinguished also on the side of the processes and functions which the given anatomical structures exercise. Medical practitioners and medical informaticians are primarily interested in structures, functions and processes at the coarser levels of granularity; theoretical biologists and bioinformaticians in those at the finer levels of granularity. We believe that in bridging the gap between the two groups of disciplines, an important role can be played by a theory of granular levels. Such a theory would be able to do justice, for example, to the fact that, even though our skin loses hundreds of thousands of cells per day, it still, at a coarser level of granularity, remains intact as an anatomical entity. It would also enable us to do justice to the fact that compounds like carcinoembryonic antigens serve as markers for colon carcinoma, or to the fact that methotrexate acts upon individual cancer cells to produce effects at the organ level. In the daily practice of a physician, such molecular interactions are not of central importance; for the biologist, however, it is precisely phenomena at such finer levels of granularity which are of principal interest. If the effective integration of life-science data is to be achieved, then we need to do justice within a single framework to all the different disciplines involved, and this means also to the associated levels of granularity.

It is not only different disciplines but also different terminology, ontology and database systems that deal with entities at different levels of granularity. But there are notorious incompatibilities both within and across such systems, and in what follows we will provide some examples designed to show how a rigorous theory of granularity can be of use in helping to eliminate some of those incompatibilities.

### Levels of granularity in anatomy

### Preliminary observations

We shall deal here with those anatomical levels of granularity which are ranged between the level of the single biological macromolecule and of the whole organism. There are lower (submolecular) levels of granularity and also levels existing above the organism (such as populations and species), but we do not consider these here. Also, not all the

Copyright © 2004 John Wiley & Sons, Ltd.

levels of granularity here discussed exist within every species. For the sake of simplicity, we focus here only on human beings.

We start with some basic principles, which we believe can serve as axioms of a full-fledged theory of granularity. These basic principles are formulated first in an unrestricted form. Later we shall see different ways in which they need to be modified to deal with certain special cases (for details on a more general framework for granularity, see Bittner and Smith, 2002):

1. Each level of granularity is determined by a class or type of *grain*.
2. The grains in a given level are parts of the grains in the next higher level.
3. Every level of granularity is such that summing all the grains together yields the entire human body.
4. The grains in a given level do not need to be all of the same size, neither do they need to be homogeneous.
5. The grains in a given level must be smaller in size than those entities on the next higher level of which they are parts.
6. With each level of granularity there is associated some specific type of causal understanding and thus some specific family of causal laws; when one moves up a level, then the grains on the lower levels become causally irrelevant.
7. Some entities can change through time in such a way that one and the same entity (an embryo, a tumour, an organism) can occupy a sequence of different levels of granularity in succession.

From principles 4 and 5, it follows that size is a criterion for drawing dividing lines between granular levels. This criterion, however, cannot be applied indiscriminately to all the entities on a given level; rather, it must be applied to each entity or group of entities in succession. For each specific entity, it holds that it is smaller in size than the grain in which it is included as part in the next higher level and larger in size than the (typically many) grains it will include as parts in the next level down.

### Preliminary identification of granular levels

Principle 1 tells us that each level of granularity is determined by a class of grains of a certain sort. It is, however, not a trivial task to determine what are the classes of grains from which the human body is built and to figure out what principles and conditions such grains must satisfy. Must all grains be maximally self-connected relative to some condition, such as being made up of cells or molecules of the same type? The example of the endocrine system tells us that it will be difficult to hold onto this principle if we allow organ systems as grains forming a level of granularity of their own (Smith *et al*., 2004). Here, we put such questions aside and simply suggest that there are the following more or less well-defined levels of granularity within the human organism (the section that follows, however, can be also taken as a preliminary justification of our list):

1. *Organism*, e.g. human body taken as a whole.
2. *Organ system*, e.g. respiratory system, digestive system.
3. *Cardinal body part*, e.g. head, thorax, abdomen.
4. *Organ*, e.g. liver, lung, kidney.
5. *Organ part*, e.g. upper lobe of lung, renal pelvis.
6. *Tissue*, e.g. pulmonary alveolar epithelium, mesothelium of pleura.
7. *Tissue subdivision*, e.g. anterior epithelium of iris.
8. *Collection of cells*, e.g. portion of menstrual secretion.
9. *Cell*, e.g. neuron, nephron, white blood cell.
10. *Collection of subcellular organelles*, e.g. rough endoplasmic reticulum, flagellar structure.
11. *Subcellular organelle*, e.g. nucleus, ribosome.
12. *Biological macromolecule*, e.g. protein, polysaccharide.

### Granularity and the mass/count distinction

In order to decide on which levels of granularity there are we must consider the distinction between count nouns such as ‘cow’, ‘suitcase’, ‘chair’, and mass nouns such as ‘beef’, ‘luggage’, ‘furniture’ (Pelletier 1991). Count nouns are nouns referring to entities — grains — that can be counted. A normal human body has two lungs, one liver, millions of cells. Mass nouns are those nouns which refer to stuff, such as blood or urine, conceived in a

Copyright © 2004 John Wiley & Sons, Ltd. 

way that traces over its granular constituents. One cannot count blood or urine, although one can count *portions* of blood or urine, including the maximal portions of these substances which exist in given containers at given times. (To speak of 'three waters' or 'four bloods' is a clumsy way of referring to *types* of water or blood; but types are precisely not parts of any particular human organism.)

Within a human body, there is at every given time one maximal portion of hepatic tissue, which is the combination of all the portions of hepatic tissue within the body at that time. Within standard anatomy, however, the mass/count distinction is not fully respected, above all as concerns the use of the words 'substance' and 'tissue'. We shall here circumvent this problem by taking tissue-terms as count-nouns referring to portions of tissue. Such portions of tissue belong in each case to specific organ parts, e.g. the maximal portions of epithelial tissue of the liver are proper parts of the hepatic lobules.

On each level of granularity the grains are marked by their own characteristic kinds of structure. Organs, cells, molecules have such characteristic structures, and so do portions of tissue and collections of subcellular organelles. Each separate portion of hepatic tissue is in a certain sense a lump, yet it has well-defined hexagonal lobules. Similarly, each portion of muscular tissue has fascicles and motor units. Our levels of granularity are now selected by paying attention to the factor of having grains, each in such a way as to satisfy grain-specific causal principles.

The different biomedical disciplines then lend different degrees of importance to the structures and causal powers of entities within different levels of granularity. Thus, while for biologists and bioinformaticians portions of tissue are in the majority of cases the coarsest structures with which they have to deal, for medical practitioners and medical informaticians the structures of primary interest go all the way up to the organ and organism levels of granularity.

Further confirmation of our proposed list of levels comes from the existing life-science disciplinary divisions both within medicine and between medicine and other disciplines, such as molecular biology, genetics, pharmacology, and so on.

### Granularity and parthood

Two levels of granularity in our list involve grains which overlap in the mereological sense of sharing common parts, implying the failure of unrestricted principles 2 and 5: the levels of cardinal body parts and of organ systems. Thus, part of the respiratory system is in the head, another part is within the chest. The two levels in question reflect partitions of the body which are skew to each other. The head, however, is listed as a grain on the level of granularity of cardinal body parts.

Apart from this example, however, the entities at the finer levels of granularity here considered are always parts of corresponding entities belonging to coarser levels of granularity, just as entities at coarser levels are associated always with entities belonging at finer levels as their parts. We represent the issue formally as follows:

Let GRAN = $\langle G_1, G_2, G_3 \ldots . G_{12} \rangle$ be the set of levels of granularity as established along the lines indicated above, ordered from coarsest to finest, and let U be the set of biological universals (e.g. those defined in GO or FMA). We define **gr** as the function of U onto GRAN. This function associates each universal with its level of granularity:

$$\mathbf{gr}{:}u \rightarrow \mathrm{gr}(u), \text{ for } u \in \mathrm{U} \text{ and } \mathrm{gr}(u) \in \mathrm{GRAN}$$

Notice that for the sake of simplicity we do not take time into consideration. This, however, will be necessary when we come to deal with entities which change their level of granularity according to their stage of development. Thus, for instance, a tumour or a human being starts as a cell or a collection of cells and gradually grows to occupy successively coarser levels of granularity.

We write '$\mathbf{inst}(x, u)$' for '$x$ is an instance of $u$', where $x$ ranges over particulars and $u$ over universals. We write '$\mathbf{part}(x, y)$', for '$x$ is a part of $y$', where $x$ and $y$ range over particulars. '∃' is the standard existential quantifier of predicate logic, and means 'there is some/there is at least one'. This enables us to assert, for example, that:

$$\begin{aligned} &\exists x\,\mathbf{inst}(x, \textit{mitochondrion}) \\ &\quad \& \ \exists y\,\mathbf{inst}(y, \ \textit{hepatocyte}) \ \& \ \mathbf{part}(x, y) \\ &\quad \& \ \mathbf{gr}(x) = G_{11}(\text{Subcellular Organelle}) \\ &\quad \& \ \mathbf{gr}(y) = G_9 \ (\text{Cell}) \end{aligned}$$

Copyright © 2004 John Wiley & Sons, Ltd. 

This means that there is some instance of mitochondrion at the subcellular organelle level of granularity which is a part of an instance of hepatocyte at the cell level. We should like, however, to assert statements to the effect that all entities of a given type at one level of granularity stand in a given relation to entities of some other given type at some other level of granularity. Unfortunately, not every instance of *mitochondrion* is a part of some instance of *hepatocyte*, since mitochondria are present in almost all human cells. We can, however, define a class consisting of all and only those mitochondria which are present within hepatocytes and call this class 'hepatocyte mitochondrion'. Using '∀' as the standard universal quantifier (meaning: for all/given any), we could then write:

$$\forall x\,\mathbf{inst}(x, \textit{hepatocyte mitochondrion}) \rightarrow \exists y\,\mathbf{inst}(y, \textit{hepatocyte})\ \&\ \mathbf{part}(x, y)\ \&\ \mathbf{gr}(x) = G_{11}\ \text{(Subcellular Organelle)}\ \&\ \mathbf{gr}(y) = G_9\ \text{(Cell)}$$

$$\forall x\,\mathbf{inst}(x, \textit{hepatocyte}) \rightarrow \exists y\,\mathbf{inst}(y, \textit{hepatocyte mitochondrion})\ \&\ \mathbf{part}(y, x)\ \&\ \mathbf{gr}(x) = G_9\ \text{(Cell)}\ \&\ \mathbf{gr}(y) = G_{11}\ \text{(Subcellular Organelle)}$$

This, however, is not an ideal solution as we move down the granular hierarchy. Thus, if we wish to refer, for instance, to the lipopolysaccharides present within the mitochondrial membrane present within hepatocytes, then the corresponding class would need to be called *hepatocyte mitochondrion membrane lipopolysaccharides*, and this expression is marked by an obvious ambiguity. We could remove the threat of ambiguity here by introducing special operators, such as '*', to pick out just those instances of a given universal which are parts of another universal. Thus, while not all mitochondria are parts of hepatocytes, we do have, trivially:

$$\textit{hepatocyte} * \textit{mitochondrion part_of hepatocyte}$$

where *hepatocyte* ∗ *mitochondrion* is that class whose instances consist of those mitochondria which are parts of hepatocytes (for an alternative approach, see Smith and Rosse, 2004).

Instances at coarser levels of granularity have instances at finer levels of granularity as parts, and instances at finer levels are parts of instances at coarser levels. We can state these principles as follows:

$$\forall x \forall u\,\mathbf{inst}(x, u)\ \&\ \mathbf{gr}(u) = G_{\mathrm{n}}\ \&\ \mathrm{n} > 1 \rightarrow \exists y \exists v\,\mathbf{inst}(y, v)\ \&\ \mathbf{gr}(v) = G_{\mathrm{n-1}}\ \&\ \mathbf{part}(x, y)$$

which means that for each instance of each universal existing at a level of granularity lower than the highest level, there is an instance of a universal at some higher (coarser) level of which the given instance is a part. The converse, where we set $G_{\mathrm{k}}$ to be the lowest level of granularity, is also true:

$$\forall x \forall u\,\mathbf{inst}(x, u)\ \&\ \mathbf{gr}(u) = G_n\ \&\ \mathrm{n} < \mathrm{k} \rightarrow \exists y \exists v\,\mathbf{inst}(y, v)\ \&\ \mathbf{gr}(v) = G_{n+1}\ \&\ \mathbf{part}(y, x)$$

For each instance of a universal at some level of granularity higher than the lowest level, there is an instance of a universal at some lower (finer) level that is a part thereof.

### Granularity and the Gene Ontology

The Gene Ontology (GO website; for a critical overview see Smith *et al.*, 2003) consists of three orthogonal axes, pertaining to cellular components, molecular functions and biological processes, respectively. GO's cellular component axis, which is the counterpart of anatomy in other biological ontologies, comprehends the universal *cell* at its highest level of granularity. There is, however, a further, more fragmentary anatomy ontology embedded within GO among the children of *development* (in terms like *fat body development* and so forth). Molecular functions are defined by GO as 'the (actual or potential) biochemical activity of a gene product' (Gene Ontology Consortium 2000). Biological processes are 'objective(s) to which the gene or gene product contributes' (*ibid.*).

Functions and processes are entities dependent on certain other entities which are their bearers (Husserl, 1900–1901; Simons, 1987). Since the independent entities recognized by GO terms in their own right have *cell* as their highest granularity, any biological process which occurs at granularities coarser than this is unable to receive an adequate representation within the GO framework,

Copyright © 2004 John Wiley & Sons, Ltd. 

since we have no way of referring to the independent entity which is its bearer.

It is clear that biological processes such as *behaviour, response to extracellular stimulus, sex determination*, etc., have component processes at the cellular level. However, they also have component processes e.g. at the organ or body system levels, and GO again lacks the means for representing these. This is not a criticism of GO: it reflects a design choice, taken by the original authors of GO, with its own pragmatic motivations. However, it does draw attention to the need to embed GO within a larger framework within which this built-in expressive paucity can be surmounted.

Another feature of the terms in the Gene Ontology related to the phenomenon of granularity is the definition of GO's *extracellular*. This term in GO's cellular component ontology is defined as meaning the space external to the outermost structure of a cell. Since no external limit is set for this external space, it could in principle extend to include all the space within the human body that is not inside a given cell. This problem could be avoided by taking the relevant size of spatial regions to be determined by the grains (i.e. by cells, in this example) associated with each of GO's constituent ontologies.

In general, GO does not provide links between its three axes, although efforts are under way to fill in this gap and GO may introduce such links at some time in the future. Within the current version, however, the relationships are not represented, which leads to the problems indicated above.

In approaching the problem of establishing links between GO's three axes, we have encountered problems especially where biological processes needed to be linked to cellular components. This is because too many cellular component terms are picked out by automatic and semi-automatic methods as eligible bearers of biological processes such as *growth, metabolism, homeostasis*, and so forth. The need to solve this problem is one of the many reasons why projects like GO should address issues of granularity in more detail and with more precision in order to enable the fine-tuning of such methods.

With the exceptions of *sensu*-terms, such as 'cytosolic ribosome (*sensu* Bacteria)', GO terms are designed to apply across all species. This, too, reflects a deliberate design choice, motivated by the desire to serve the cross-species annotation of genes and gene products. It has the disadvantage, however, that it becomes difficult to understand the meaning behind those GO terms like *adult behavior*, which would paradigmatically refer to human beings, but which could also, for example, refer to unicellular organisms when applied to entities at the cellular level of granularity.

One way to get round this problem is to take into account the existing species-specific annotations of gene products to terms in GO's three axes. Thus, just as we can create extra-ontological links between terms in GO's three separate axes by looking at the ways in which such terms are used in annotations of the same genes or gene products, so we could use the ways in which ambiguous terms are used in annotations to species at different levels of granularity in order to ensure univocal interpretations.

### Granularity and the Foundational Model of Anatomy

The Foundational Model of Anatomy (FMA) (FMA website; Mejino *et al*., 2003; Rosse and Mejino, 2003; Smith and Rosse, 2004) comprehends all but two of the levels of granularity mentioned in our list above. The exceptions are: *collection of cells* and *collection of subcellular organelles*. Levels of granularity are not included in the FMA as such. Rather, each is represented by a specific anatomical universal, so that the universals belonging to each level stand in a subsumption relationship to the universal marking the level in question.

To treat granularity in terms of subsumption in this way is once again a simple design choice. One disadvantage of the approach, however, is that the ontological zooming between levels of granularity, e.g. of the sort which occurs when we move from viewing a tumour in terms of molecular structures to viewing it in terms of cellular structures, can be more easily captured by software tools when the levels of granularity are *explicitly* distinguished (Bittner and Smith, 2002). The relations which obtain between entities belonging to different levels of granularity, e.g. the cross-granular parthood relation between *superficial layer of corneal epithelium* (or *deep layer of corneal epithelium*) and *corneal epithelium*, are not always explicitly represented as such within the FMA.

Copyright © 2004 John Wiley & Sons, Ltd. 

### Granularity and SNOMED CT

SNOMED CT (SNOMED CT website) includes the class *anatomical structure*, which subsumes in its turn subclasses corresponding to what we have been here calling levels of granularity, and they cover some of the levels of granularity in this way. Unfortunately, the SNOMED children of the class *anatomical structure* are not pairwise disjoint. Anatomical structure in SNOMED is classified as a physical anatomical entity and its subclasses include *developmental body structure, intercellular anatomical structure, entire anatomical structure, transplant, structure of product of conception, body region structure, sex structure, body system structure, body tissue structure, body organ structure, body wall structure* and *cell structure.*

*Sex structure, structure of product of conception, transplant* and *developmental body structure* overlap with *body organ structure* and *body tissue structure* and, indeed, with each other, in a way which should be avoided in a robust ontology.

### The price of neglecting levels of granularity

Some of the problems which occur when we neglect levels of granularity are as follows:

1. The treatment of anatomical entities as entities which maintain their identity through time is hampered. Data integration hitherto has been constrained to work with data in which attributes are assigned primarily to entities at some one specific level of granularity. Stronger data integration frameworks can be constructed if we have explicit tools for keeping track of entities and their attributes as they traverse different levels.
2. An explicit representation along the lines presented above would enable us to do justice to the fact that, for example, cellular development is different from development of lungs, even if both are types of development.
3. Some representation of granularity is needed also to represent the fact that, for example, the infection of only one cell within the lung is not pneumonia.
4. If one cell in the colon epithelium has a p53 mutation, then since the cell is a part of the colon and since the p53 mutation is associated with colon carcinoma, a computer could falsely infer that colon carcinoma exists. Similarly, a haemorrhage in one pulmonary arteriole can be without any systemic effects. Thus, we need to distinguish this case from the case of a haemorrhage of the lung. Without some representation of levels of granularity, however, a haemorrhage involving one full lobe of the lung would not be distinguishable from a haemorrhage involving only one arteriole. Similarly, the loss of even hundreds of thousands of cells from the mucosa of the gastrointestinal tract should not lead to the inference of a generalized ulcer of the tract, and the death of hundreds of cells within the uterine epithelium should not lead to the inference of uterine necrosis.
5. Terms such as 'development' need to be used in such a way that we can distinguish formally between, for example, development in a unicellular bacterium and development of social behaviour in human beings.
6. Some cells within the heart might secrete endocrinal products. This would not, however, make the heart an endocrine organ.

### Granularity of functions and processes

It is not a trivial exercise to represent granular levels within anatomy. But representing such levels within the dimensions of function and process is an even more difficult task. A *function* is something like a potential to act in a certain way. Functions are continuant entities, which means that they preserve their identity through time from one realization to the next (and also that they can exist even when they are not being realized at all). The realizations of functions are *processes*, e.g. of respiration. This means then that such realizations are entities whose instances occur through a period of time and are not present in totality at any particular instant of time. Processes can be segmented into temporal parts (e.g. into a beginning, a middle, and an end). Both functions and processes are dependent entities, which means that they cannot exist without being realized in or by some underlying continuant bearer or bearers, e.g. a cell, an organelle, a human being.

There are three ways to represent the granularity of functions and processes: (a) on the basis

Copyright © 2004 John Wiley & Sons, Ltd.

of the granularity levels of their underlying bearers; (b) on the basis of time, applying to processes directly and to functions indirectly via their realizations — the analogue of size in the case of granularity for anatomical entities; and (c) on the basis of the parthood relations which exist between different functions on the one hand or between different processes on the other.

(a) From the anatomical perspective, cellular functions are dependent on the cells which are their bearers and thus exist at the cellular level of granularity; organ functions are dependent on the organs which are their bearers and thus exist at the organ level of granularity.
(b) The temporal extent of a biological process can be measured in years, months, weeks, days, hours, minutes, seconds and so on. Processes, including the realizations of functions, have coarser or a finer grain from the temporal perspective according to whether they take a longer or shorter time.
(c) The most involved perspective on granularity for processes or functions conceives the latter in terms of chains of parthood relations obtaining between the processes or functions involved. Unfortunately, this method does not yield a clear ordering of levels, since it is not clear when processes do or do not stand to each other in part relations. Does a process of respiration associated with a process of running stand to the latter as part to whole? Is regulation of sleep a part of sleep? Is ageing a part of dying? Moreover, not all instances of a smaller process or function are parts of any larger process or function, and not all instances of a larger process or function have instances of smaller processes or functions as parts. For example, *hexokinase 1* (KEGG K00844, EC 2.7.1.1) *activity* is involved not only in *glycolysis* but also in all of the following: *fructose and mannose metabolism, galactose metabolism, starch and sucrose metabolism* and *aminosugar metabolism*. In such a case, we can create terms such as:

*hexokinase 1 activity involved_in glycolytic pathway*

in order to represent those cases where hexokinase 1 activity is involved in glycolysis, or alternatively:

*hexokinase 1 activity involved_in galactose metabolism pathway*

in order to represent those instances where hexokinase 1 activity is involved in some galactose metabolism pathway, and so on.

Only when we are able to represent the instances of a function or a process which are parts of a larger function or process, can we locate them within their bearers, assign to them facilitators or inhibitors, substrates or products and so on.

### Conclusion

The fact that organisms are built out of structures organized in a granular way has long been well known to domain specialists. In this paper we have embarked upon a preliminary investigation of the phenomenon of granularity, identifying the main issues, proposing some elements of an explicit formalism and commenting on the implicit ways in which granularity has been represented in ontologies such as GO, FMA, SNOMED CT thus far. We hope to have made clear that any good reference ontology of human (and non-human) organisms needs to avail itself of tools for representing granularity in an explicit way.

### Acknowledgements

This paper was written under the auspices of the Wolfgang Paul Program of the Alexander von Humboldt Foundation, the European Union Network of Excellence on Medical Informatics and Semantic Data Mining, and the Volkswagen Foundation under the auspices of the project 'Forms of Life'.

### References

Bittner T, Smith B. 2002. A theory of granular partitions. In *Foundations of Geographic Information Science*, Duckham M, Goodchild M, Worboys M (eds). Taylor & Francis: London.
FMA website: **http://sig.biostr.washington.edu/projects/fm/AboutFM.html**
Gene Ontology Consortium. 2000. Gene Ontology: tool for the unification of biology. *Nature Genet* **25**: 25–29.
GO website: **http://www.geneontology.org**

Copyright © 2004 John Wiley & Sons, Ltd. 

Husserl E. 1900–1901. *Logical Investigations*, Findlay JN (trans). Allen & Unwin: London [the original work, *Logische Untersuchungen*, appeared in 1900, 1901].

Mejino JLV, Agoncillo AV, Rickard KL, Rosse C. 2003. Representing complexity in part–whole relationships within the Foundational Model of Anatomy. In *Proceedings, American Medical Informatics Association Fall Symposium*, 450–454.

Pelletier FJ. 1991. Mass terms. In *Handbook of Metaphysics and Ontology*, Smith B, Burkhardt H (eds). Philosophia: Munich, 495–499.

Rosse C, Mejino JLV Jr. 2003. A reference ontology for biomedical informatics: the Foundational Model of Anatomy. *J Biomed Informat* **36**: 478–500.

Simons P. 1987. *Parts: A Study in Ontology*. Clarendon Press: Oxford.

Smith B, Munn K, Papakin I. 2004. Bodily systems and the spatio-functional structure of the human body. In *Ontologies in Medicine*, Pisanelli DM (ed.). IOS Press: Amsterdam.

Smith B, Rosse C. 2004. The role of foundational relations in the alignment of biomedical ontologies. In *Proceedings of MedInfo*, 444–448.

Smith B, Williams J, Schulze-Kremer S. 2003. The ontology of the gene ontology. In *Proceedings of AMIA Symposium* 609–613.

SNOMED CT website: **http://www.snomed.org/snomedct/what_is.html**

Copyright © 2004 John Wiley & Sons, Ltd. 
